# *catena*-Poly[[[aqua­(1,10-phenanthroline-κ^2^ *N*,*N*′)manganese(II)]-{μ-4,4′-[(4-carb­oxy­benz­yl)nitrilo]­dibenzoato-κ^4^ *O*,*O*′:*O*′′,*O*′′′}] monohydrate]

**DOI:** 10.1107/S1600536812016819

**Published:** 2012-04-21

**Authors:** Jin-Song Hu, Lei Jing, Xiao-Ming Song, Jie He

**Affiliations:** a School of Chemical Engineering, Anhui University of Science and Technology, Huainan Anhui 232001, People’s Republic of China

## Abstract

The title compound, {[Mn(C_22_H_15_NO_6_)(C_12_H_8_N_2_)(H_2_O]·H_2_O}_*n*_, was obtained under solvothermal conditions. The Mn^2+^ cation exhibits a distorted penta­gonal–bipyramidal MnN_2_O_5_ coordination sphere with the water O atom and one of the phenanthroline N atoms in the axial positions. The cation is bridged by the doubly deprotonated 4,4′-[(4-carb­­oxy­benz­yl)nitrilo]­dibenzoate ligand, generating a polymeric chain parallel to [100]. O—H⋯O hydrogen bonding, as well as π–π inter­actions between neighbouring phenanthroline ligands, with centroid–centroid distances of 3.695 (1) Å, lead to the construction of a three-dimensional network.

## Related literature

For background to compounds with metal-organic-framework structures (MOFs), see: Corma *et al.* (2010 ▶); Feng *et al.* (2009 ▶); Lin *et al.* (2010 ▶); Ma *et al.* (2010 ▶); Sarma *et al.* (2011 ▶).
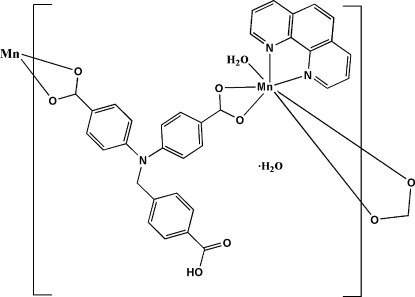


## Experimental

### 

#### Crystal data

[Mn(C_22_H_15_NO_6_)(C_12_H_8_N_2_)(H_2_O]·H_2_O
*M* *_r_* = 660.53
Monoclinic, 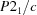

*a* = 15.142 (2) Å
*b* = 9.6734 (13) Å
*c* = 21.313 (3) Å
β = 107.445 (3)°
*V* = 2978.4 (7) Å^3^
*Z* = 4
Mo *K*α radiation
μ = 0.50 mm^−1^
*T* = 273 K
0.32 × 0.27 × 0.23 mm

#### Data collection

Bruker APEX SMART CCD diffractometer
Absorption correction: multi-scan (*SADABS*; Bruker, 2000 ▶) *T*_min_ = 0.851, *T*_max_ = 0.891
14335 measured reflections
5235 independent reflections
3488 reflections with *I* > 2σ(*I*)
*R*_int_ = 0.102

#### Refinement

*R*[*F* ^2^ > 2σ(*F* ^2^)] = 0.060
*wR*(*F* ^2^) = 0.156
*S* = 0.95
5235 reflections
410 parameters
H-atom parameters constrained
Δρ_max_ = 0.62 e Å^−3^
Δρ_min_ = −0.45 e Å^−3^

### 

Data collection: *SMART* (Bruker, 2000 ▶); cell refinement: *SAINT* (Bruker, 2000 ▶); data reduction: *SAINT*; program(s) used to solve structure: *SHELXS97* (Sheldrick, 2008 ▶); program(s) used to refine structure: *SHELXL97* (Sheldrick, 2008 ▶); molecular graphics: *DIAMOND* (Brandenburg, 2006 ▶); software used to prepare material for publication: *publCIF* (Westrip, 2010 ▶).

## Supplementary Material

Crystal structure: contains datablock(s) global, I. DOI: 10.1107/S1600536812016819/wm2618sup1.cif

Structure factors: contains datablock(s) I. DOI: 10.1107/S1600536812016819/wm2618Isup2.hkl

Supplementary material file. DOI: 10.1107/S1600536812016819/wm2618Isup3.cdx

Additional supplementary materials:  crystallographic information; 3D view; checkCIF report

## Figures and Tables

**Table 1:** Selected bond lengths (Å)

Mn1—O1	2.198 (2)
Mn1—O4^i^	2.210 (2)
Mn1—O9	2.235 (2)
Mn1—N1	2.258 (3)
Mn1—N2	2.274 (3)
Mn1—O2	2.389 (2)
Mn1—O3^i^	2.461 (3)

Symmetry code: (i) 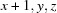.

**Table 2:** Hydrogen-bond geometry (Å, °)

*D*—H⋯*A*	*D*—H	H⋯*A*	*D*⋯*A*	*D*—H⋯*A*
O8—H8*A*⋯O4^ii^	0.85	1.96	2.798 (4)	170
O8—H8*B*⋯O11^iii^	0.85	2.43	2.872 (5)	113
O9—H9*B*⋯O1^iv^	0.85	2.09	2.745 (3)	134
O9—H9*A*⋯O3^v^	0.82	2.12	2.824 (3)	144
O12—H12⋯O8^vi^	0.82	1.81	2.605 (4)	165

Symmetry codes: (ii) 
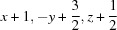
; (iii) 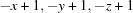; (iv) 
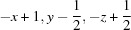
; (v) 
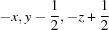
; (vi) 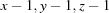.

**Fig. 1.**
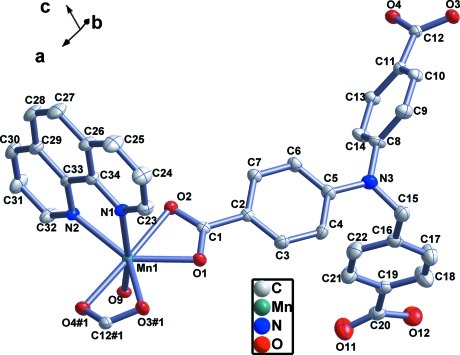
The molecular structure of the title compound, showing the coordination of the MnII cation. Displacement ellipsoids are drawn at the 20% probability level. [Symmetry codes: (#1) 1 + x, y, z.]

**Fig. 2.**
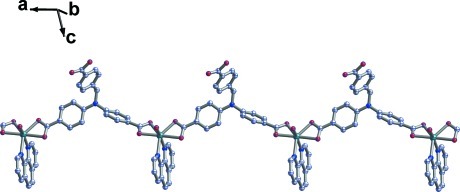
A view of the polymeric zigzag chain expanding parallel to [100]; the phen ligands are arranged in parallel fashion.

**Fig. 3.**
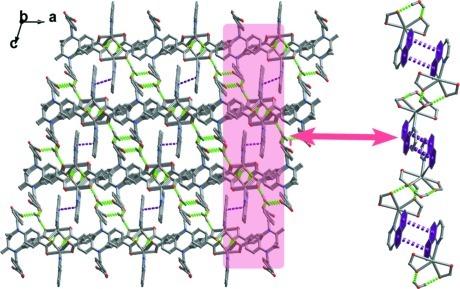
A view of the three-dimensional network formed by O–H..O hydrogen bonding (green dotted lines) and π—π interactions (purple dotted lines).

## supplementary crystallographic information

### Comment

The construction of compounds with metal-organic framework structures (MOFs) from various molecular building blocks connected by coordination bonds or supramolecular contacts have been of intense interest due to their structures and topological features (Ma *et al.*, 2010), as well as their promising applications in photochemistry areas (Feng *et al.*, 2009), molecular magnetism (Sarma *et al.*, 2011), heterogeneous catalysis (Corma *et al.*, 2010), and molecular sorption (Lin *et al.*, 2010). We recently designed and synthesized (4-carboxybenzyl)-4,4'-nitrilodibenzoic acid (H_3_*L*), a tripod carboxylate ligand. To test the ability of this ligand to give new architectures and topologies, we selected this ligand, 1,10-phenanthroline (phen), and an Mn^II^ salt to solvothermally synthesize the new coordination polymer [Mn(H*L*)(phen)(H_2_O)]^.^H_2_O or [Mn(C_22_H_15_NO_6_)(C_12_H_8_N_2_)(H_2_O]^.^H_2_O .

The asymmetric unit of the title compound contains one Mn^II^ ion, one H*L*^2-^ anion, one phen ligand and one lattice water molecule (Fig. 1) The cation displays a distorted MnN_2_O_5_ coordination sphere that can be best described as pentagonal-bipyramidal. As shown in Figure 1, the Mn^II^ cation is coordinated by two pairs of chelating carboxylate O atoms from two H*L*^2-^ ligands, one coordinating water, and two N atoms from one phen ligand. The Mn—N bond lengths are 2.258 (3) and 2.274 (3) Å, and the Mn—O lengths are in the range of 2.198 (2)–2.461 (3) Å. The dihedral angles between the three phenyl rings in the anion are 70.16 (18)°, 83.39 (19)° and 77.4 (12)°.

Neighboring Mn^II^ ions are linked by bridging H*L*^2-^ anions to form a polymeric zigzag chain parallel to [100]. The distance between adjacent Mn^II^ cation withion the chain is 15.142 (1) Å (Figure 2). O—H···O hydrogen bonding between the carboxy group and the coordinating and free water molecules as donors and carboxylate O atoms and water O atoms as acceptors (Table 2) as well as π—π interactions between neighbouring phen groups with centroid-to-centroid distances of 3.695 (1) Å stabilize the three-dimensional set-up of the structure (Figure 3).

### Experimental

A mixture of MnCl_2_^.^4H_2_O (10 mg), H3*L* (20 mg) and phen (10 mg) was dissolved in 9 ml of DMF/H2O(1:2, *v*/*v*). The final mixture was placed in a Parr Teflon-lined stainless steel vessel (15 ml) under autogenous pressure and heated at 363 K for 3 d. A large quantity of colorless crystals were obtained, which were washed with mother liquid, and dried under ambient conditions (yield: 71% based on phen).

### Refinement

The water H-atoms were located from a Fourier differnce map and were refined with distance restraintes of O—H = 0.85 (2) Å, and H—H distance of 1.45 (2) Å with *U*_iso_(H) = 1.5Ueq(O). The C-bound H-atoms were placed in geometrically idealized positions and treated as riding: C—H = 0.93 and 0.96 Å for CH and CH_2_ H-atoms, respectively, with *U*_iso_(H) = k τimes *U*_eq_(C), where k = 1.5 for CH_2_ H-atoms, and k = 1.2 for all other H-atoms. The O atom of the solvent water molecule was refined with an isotropic displacement parameter.

### Crystal data

**Table:**

[Mn(C_22_H_15_NO_6_)(C_12_H_8_N_2_)(H_2_O]·H_2_O	*F*(000) = 1364
*M**_r_* = 660.53	*D*_x_ = 1.473 Mg m^−^^3^
Monoclinic, *P*2_1_/*c*	Mo *K*α radiation, λ = 0.71073 Å
Hall symbol: -P 2ybc	Cell parameters from 2883 reflections
*a* = 15.142 (2) Å	θ = 2.3–27.9°
*b* = 9.6734 (13) Å	µ = 0.50 mm^−^^1^
*c* = 21.313 (3) Å	*T* = 273 K
β = 107.445 (3)°	Block, colorless
*V* = 2978.4 (7) Å^3^	0.32 × 0.27 × 0.23 mm
*Z* = 4	

### Data collection

**Table:**

Bruker APEX SMART CCD diffractometer	5235 independent reflections
Radiation source: fine-focus sealed tube	3488 reflections with *I* > 2σ(*I*)
Graphite monochromator	*R*_int_ = 0.102
φ and ω scans	θ_max_ = 25.0°, θ_min_ = 2.0°
Absorption correction: multi-scan (*SADABS*; Bruker, 2000)	*h* = −18→17
*T*_min_ = 0.851, *T*_max_ = 0.891	*k* = −11→11
14335 measured reflections	*l* = −25→15

### Refinement

**Table:**

Refinement on *F*^2^	Primary atom site location: structure-invariant direct methods
Least-squares matrix: full	Secondary atom site location: difference Fourier map
*R*[*F*^2^ > 2σ(*F*^2^)] = 0.060	Hydrogen site location: inferred from neighbouring sites
*wR*(*F*^2^) = 0.156	H-atom parameters constrained
*S* = 0.95	*w* = 1/[σ^2^(*F*_o_^2^) + (0.0787*P*)^2^] where *P* = (*F*_o_^2^ + 2*F*_c_^2^)/3
5235 reflections	(Δ/σ)_max_ = 0.001
410 parameters	Δρ_max_ = 0.62 e Å^−^^3^
0 restraints	Δρ_min_ = −0.45 e Å^−^^3^

### Special details

**Table:**

Geometry. All e.s.d.'s (except the e.s.d. in the dihedral angle between two l.s. planes) are estimated using the full covariance matrix. The cell e.s.d.'s are taken into account individually in the estimation of e.s.d.'s in distances, angles and torsion angles; correlations between e.s.d.'s in cell parameters are only used when they are defined by crystal symmetry. An approximate (isotropic) treatment of cell e.s.d.'s is used for estimating e.s.d.'s involving l.s. planes.
Refinement. Refinement of *F*^2^ against ALL reflections. The weighted *R*-factor *wR* and goodness of fit *S* are based on *F*^2^, conventional *R*-factors *R* are based on *F*, with *F* set to zero for negative *F*^2^. The threshold expression of *F*^2^ > σ(*F*^2^) is used only for calculating *R*-factors(gt) *etc*. and is not relevant to the choice of reflections for refinement. *R*-factors based on *F*^2^ are statistically about twice as large as those based on *F*, and *R*- factors based on ALL data will be even larger.

### Fractional atomic coordinates and isotropic or equivalent isotropic displacement parameters (Å^2^)

**Table:**

	*x*	*y*	*z*	*U*_iso_*/*U*_eq_	
C1	0.3740 (2)	0.7528 (3)	0.29214 (19)	0.0385 (8)	
C2	0.2736 (2)	0.7716 (3)	0.25997 (18)	0.0412 (8)	
C3	0.2389 (3)	0.7855 (4)	0.1934 (2)	0.0543 (10)	
H3	0.2792	0.7920	0.1681	0.065*	
C4	0.1436 (3)	0.7902 (4)	0.1629 (2)	0.0606 (11)	
H4	0.1210	0.7955	0.1173	0.073*	
C5	0.0817 (2)	0.7869 (4)	0.2000 (2)	0.0528 (10)	
C6	0.1170 (2)	0.7801 (4)	0.2663 (2)	0.0531 (10)	
H6	0.0773	0.7823	0.2922	0.064*	
C7	0.2110 (2)	0.7702 (3)	0.2959 (2)	0.0478 (9)	
H7	0.2332	0.7622	0.3415	0.057*	
C8	−0.0824 (3)	0.7829 (4)	0.2049 (2)	0.0564 (10)	
C9	−0.1346 (3)	0.8984 (4)	0.2073 (2)	0.0589 (11)	
H9	−0.1226	0.9813	0.1893	0.071*	
C10	−0.2044 (3)	0.8919 (4)	0.2363 (2)	0.0524 (10)	
H10	−0.2394	0.9702	0.2375	0.063*	
C11	−0.2229 (2)	0.7697 (3)	0.26370 (17)	0.0415 (8)	
C12	−0.3046 (3)	0.7581 (4)	0.28836 (19)	0.0469 (9)	
C13	−0.1686 (2)	0.6567 (4)	0.2626 (2)	0.0511 (10)	
H13	−0.1787	0.5747	0.2822	0.061*	
C14	−0.0992 (3)	0.6628 (4)	0.2329 (2)	0.0609 (11)	
H14	−0.0639	0.5847	0.2319	0.073*	
C15	−0.0547 (3)	0.7657 (4)	0.1000 (2)	0.0675 (12)	
H15A	−0.0172	0.8111	0.0764	0.081*	
H15B	−0.1161	0.8062	0.0855	0.081*	
C16	−0.0618 (3)	0.6100 (4)	0.0825 (2)	0.0592 (11)	
C17	−0.1327 (3)	0.5656 (5)	0.0317 (3)	0.0939 (17)	
H17	−0.1743	0.6293	0.0064	0.113*	
C18	−0.1442 (3)	0.4278 (5)	0.0170 (3)	0.099 (2)	
H18	−0.1964	0.3987	−0.0160	0.119*	
C19	−0.0806 (3)	0.3322 (4)	0.0497 (2)	0.0566 (10)	
C20	−0.0882 (3)	0.1837 (5)	0.0329 (2)	0.0652 (12)	
C21	−0.0069 (3)	0.3772 (5)	0.0987 (2)	0.0753 (13)	
H21	0.0383	0.3148	0.1211	0.090*	
C22	0.0016 (3)	0.5167 (4)	0.1156 (2)	0.0707 (13)	
H22	0.0517	0.5462	0.1502	0.085*	
C23	0.5734 (3)	1.0407 (4)	0.3660 (2)	0.0578 (10)	
H23	0.5594	1.0478	0.3205	0.069*	
C24	0.5840 (3)	1.1605 (4)	0.4018 (3)	0.0762 (14)	
H24	0.5780	1.2461	0.3810	0.091*	
C25	0.6034 (4)	1.1522 (4)	0.4681 (3)	0.0815 (15)	
H25	0.6106	1.2325	0.4931	0.098*	
C26	0.6127 (3)	1.0233 (4)	0.4988 (2)	0.0615 (11)	
C27	0.6320 (3)	1.0064 (5)	0.5675 (2)	0.0796 (14)	
H27	0.6385	1.0839	0.5943	0.096*	
C28	0.6411 (3)	0.8794 (5)	0.5946 (2)	0.0787 (14)	
H28	0.6543	0.8709	0.6399	0.094*	
C29	0.6310 (3)	0.7583 (4)	0.55564 (19)	0.0551 (10)	
C30	0.6373 (3)	0.6263 (5)	0.5807 (2)	0.0684 (12)	
H30	0.6486	0.6131	0.6256	0.082*	
C31	0.6274 (4)	0.5170 (5)	0.5410 (2)	0.0806 (15)	
H31	0.6317	0.4276	0.5577	0.097*	
C32	0.6105 (3)	0.5400 (4)	0.4740 (2)	0.0673 (12)	
H32	0.6062	0.4634	0.4469	0.081*	
C33	0.6116 (2)	0.7712 (4)	0.48698 (16)	0.0406 (8)	
C34	0.6021 (2)	0.9068 (3)	0.45838 (18)	0.0439 (9)	
Mn1	0.55471 (3)	0.71229 (5)	0.33754 (2)	0.03684 (19)	
N1	0.58186 (19)	0.9152 (3)	0.39227 (14)	0.0423 (7)	
N2	0.60042 (19)	0.6619 (3)	0.44679 (14)	0.0415 (7)	
N3	−0.0154 (2)	0.7910 (4)	0.16835 (18)	0.0635 (9)	
O1	0.43035 (16)	0.7798 (2)	0.26018 (12)	0.0466 (6)	
O2	0.40297 (16)	0.7085 (2)	0.34972 (13)	0.0504 (6)	
O3	−0.37284 (18)	0.8342 (3)	0.26431 (14)	0.0684 (8)	
O4	−0.30488 (16)	0.6694 (3)	0.33120 (14)	0.0535 (7)	
O8	0.8366 (2)	0.9035 (3)	0.94467 (16)	0.0802 (9)*	
H8A	0.7988	0.8752	0.9089	0.096*	
H8B	0.8773	0.8417	0.9599	0.120*	
O9	0.52533 (15)	0.4896 (2)	0.31171 (12)	0.0470 (6)	
H9B	0.5622	0.4606	0.2914	0.056*	
H9A	0.4717	0.4808	0.2883	0.070*	
O11	−0.0275 (3)	0.1014 (3)	0.0524 (2)	0.1007 (13)	
O12	−0.1701 (2)	0.1488 (3)	−0.00651 (18)	0.0895 (11)	
H12	−0.1699	0.0666	−0.0158	0.134*	

### Atomic displacement parameters (Å^2^)

**Table:**

	*U*^11^	*U*^22^	*U*^33^	*U*^12^	*U*^13^	*U*^23^
C1	0.041 (2)	0.0294 (17)	0.047 (2)	−0.0021 (14)	0.0158 (18)	−0.0031 (16)
C2	0.041 (2)	0.0359 (19)	0.046 (2)	−0.0024 (15)	0.0128 (18)	−0.0018 (17)
C3	0.053 (2)	0.067 (3)	0.046 (2)	−0.002 (2)	0.018 (2)	−0.001 (2)
C4	0.057 (3)	0.078 (3)	0.040 (2)	0.001 (2)	0.003 (2)	0.001 (2)
C5	0.039 (2)	0.054 (2)	0.066 (3)	−0.0064 (18)	0.018 (2)	0.000 (2)
C6	0.043 (2)	0.059 (2)	0.060 (3)	−0.0049 (18)	0.019 (2)	0.003 (2)
C7	0.043 (2)	0.049 (2)	0.054 (2)	−0.0046 (17)	0.0185 (19)	0.0010 (18)
C8	0.059 (2)	0.056 (2)	0.067 (3)	0.003 (2)	0.040 (2)	0.000 (2)
C9	0.069 (3)	0.051 (2)	0.067 (3)	−0.001 (2)	0.035 (2)	0.009 (2)
C10	0.054 (2)	0.046 (2)	0.061 (3)	0.0094 (17)	0.023 (2)	−0.0004 (19)
C11	0.042 (2)	0.044 (2)	0.038 (2)	0.0005 (16)	0.0124 (17)	−0.0012 (17)
C12	0.045 (2)	0.054 (2)	0.042 (2)	0.0016 (17)	0.0130 (18)	−0.0084 (19)
C13	0.053 (2)	0.045 (2)	0.063 (3)	0.0002 (18)	0.029 (2)	0.0066 (19)
C14	0.067 (3)	0.047 (2)	0.084 (3)	0.0106 (19)	0.045 (2)	0.007 (2)
C15	0.059 (3)	0.080 (3)	0.063 (3)	−0.001 (2)	0.018 (2)	0.004 (2)
C16	0.063 (3)	0.064 (3)	0.057 (3)	0.003 (2)	0.027 (2)	0.000 (2)
C17	0.068 (3)	0.086 (4)	0.110 (5)	0.004 (3)	0.001 (3)	−0.013 (3)
C18	0.069 (3)	0.077 (4)	0.136 (6)	−0.002 (3)	0.007 (3)	−0.036 (3)
C19	0.060 (3)	0.061 (3)	0.055 (3)	−0.011 (2)	0.027 (2)	−0.009 (2)
C20	0.065 (3)	0.073 (3)	0.060 (3)	−0.011 (2)	0.022 (2)	−0.016 (2)
C21	0.082 (3)	0.072 (3)	0.064 (3)	−0.001 (2)	0.010 (3)	−0.007 (3)
C22	0.071 (3)	0.069 (3)	0.062 (3)	−0.016 (2)	0.004 (2)	−0.016 (2)
C23	0.075 (3)	0.047 (2)	0.054 (3)	−0.001 (2)	0.024 (2)	0.005 (2)
C24	0.112 (4)	0.037 (2)	0.081 (4)	−0.006 (2)	0.032 (3)	0.001 (2)
C25	0.125 (4)	0.043 (3)	0.073 (4)	−0.006 (3)	0.024 (3)	−0.015 (3)
C26	0.082 (3)	0.055 (3)	0.047 (3)	−0.006 (2)	0.018 (2)	−0.015 (2)
C27	0.110 (4)	0.074 (3)	0.049 (3)	−0.011 (3)	0.015 (3)	−0.027 (3)
C28	0.100 (4)	0.095 (4)	0.033 (2)	−0.006 (3)	0.008 (2)	−0.015 (3)
C29	0.063 (3)	0.065 (3)	0.036 (2)	−0.003 (2)	0.013 (2)	−0.003 (2)
C30	0.086 (3)	0.079 (3)	0.037 (2)	0.001 (3)	0.015 (2)	0.015 (2)
C31	0.125 (4)	0.061 (3)	0.051 (3)	0.001 (3)	0.019 (3)	0.020 (2)
C32	0.112 (4)	0.045 (2)	0.044 (3)	0.003 (2)	0.022 (2)	0.001 (2)
C33	0.042 (2)	0.050 (2)	0.0301 (19)	−0.0005 (16)	0.0098 (16)	−0.0036 (17)
C34	0.049 (2)	0.044 (2)	0.037 (2)	−0.0039 (16)	0.0098 (18)	−0.0060 (17)
Mn1	0.0429 (3)	0.0387 (3)	0.0309 (3)	0.0016 (2)	0.0141 (2)	−0.0008 (2)
N1	0.0543 (18)	0.0370 (16)	0.0385 (18)	−0.0025 (13)	0.0183 (15)	0.0005 (13)
N2	0.0516 (18)	0.0366 (16)	0.0367 (17)	0.0020 (13)	0.0141 (14)	0.0019 (14)
N3	0.054 (2)	0.083 (3)	0.060 (2)	−0.0020 (18)	0.0272 (18)	0.0002 (19)
O1	0.0400 (14)	0.0557 (15)	0.0485 (15)	0.0001 (11)	0.0202 (12)	0.0087 (12)
O2	0.0480 (15)	0.0593 (16)	0.0463 (17)	0.0040 (12)	0.0176 (13)	0.0083 (13)
O3	0.0496 (17)	0.101 (2)	0.0576 (19)	0.0240 (15)	0.0211 (14)	0.0105 (17)
O4	0.0480 (15)	0.0631 (16)	0.0564 (17)	0.0011 (12)	0.0260 (13)	0.0070 (14)
O9	0.0466 (14)	0.0496 (14)	0.0474 (15)	−0.0012 (11)	0.0180 (12)	−0.0133 (12)
O11	0.094 (3)	0.069 (2)	0.120 (3)	0.0039 (19)	0.002 (2)	−0.022 (2)
O12	0.081 (2)	0.076 (2)	0.105 (3)	−0.0160 (17)	0.018 (2)	−0.027 (2)

### Geometric parameters (Å, º)

**Table:**

C1—O2	1.249 (4)	C21—C22	1.394 (5)
C1—O1	1.268 (4)	C21—H21	0.9300
C1—C2	1.480 (5)	C22—H22	0.9300
C2—C3	1.363 (5)	C23—N1	1.327 (4)
C2—C7	1.387 (5)	C23—C24	1.370 (6)
C3—C4	1.395 (5)	C23—H23	0.9300
C3—H3	0.9300	C24—C25	1.357 (6)
C4—C5	1.397 (6)	C24—H24	0.9300
C4—H4	0.9300	C25—C26	1.396 (6)
C5—C6	1.354 (6)	C25—H25	0.9300
C5—N3	1.423 (5)	C26—C34	1.398 (5)
C6—C7	1.376 (5)	C26—C27	1.413 (6)
C6—H6	0.9300	C27—C28	1.347 (6)
C7—H7	0.9300	C27—H27	0.9300
C8—C14	1.364 (5)	C28—C29	1.417 (6)
C8—C9	1.380 (5)	C28—H28	0.9300
C8—N3	1.454 (5)	C29—C30	1.377 (6)
C9—C10	1.376 (5)	C29—C33	1.409 (5)
C9—H9	0.9300	C30—C31	1.334 (6)
C10—C11	1.384 (5)	C30—H30	0.9300
C10—H10	0.9300	C31—C32	1.390 (6)
C11—C13	1.372 (5)	C31—H31	0.9300
C11—C12	1.486 (5)	C32—N2	1.302 (4)
C12—O3	1.247 (4)	C32—H32	0.9300
C12—O4	1.254 (4)	C33—N2	1.340 (4)
C13—C14	1.381 (5)	C33—C34	1.436 (5)
C13—H13	0.9300	C34—N1	1.352 (4)
C14—H14	0.9300	Mn1—O1	2.198 (2)
C15—N3	1.420 (5)	Mn1—O4^i^	2.210 (2)
C15—C16	1.548 (6)	Mn1—O9	2.235 (2)
C15—H15A	0.9700	Mn1—N1	2.258 (3)
C15—H15B	0.9700	Mn1—N2	2.274 (3)
C16—C17	1.346 (6)	Mn1—O2	2.389 (2)
C16—C22	1.352 (5)	Mn1—O3^i^	2.461 (3)
C17—C18	1.368 (6)	O3—Mn1^ii^	2.461 (3)
C17—H17	0.9300	O4—Mn1^ii^	2.210 (2)
C18—C19	1.367 (6)	O8—H8A	0.8501
C18—H18	0.9300	O8—H8B	0.8500
C19—C21	1.351 (5)	O9—H9B	0.8501
C19—C20	1.477 (6)	O9—H9A	0.8200
C20—O11	1.193 (5)	O12—H12	0.8199
C20—O12	1.316 (5)		
			
O2—C1—O1	120.3 (3)	C25—C24—C23	118.8 (4)
O2—C1—C2	120.4 (3)	C25—C24—H24	120.6
O1—C1—C2	119.2 (3)	C23—C24—H24	120.6
C3—C2—C7	117.5 (3)	C24—C25—C26	120.1 (4)
C3—C2—C1	121.1 (4)	C24—C25—H25	119.9
C7—C2—C1	121.4 (3)	C26—C25—H25	119.9
C2—C3—C4	120.7 (4)	C25—C26—C34	117.0 (4)
C2—C3—H3	119.6	C25—C26—C27	123.3 (4)
C4—C3—H3	119.6	C34—C26—C27	119.7 (4)
C3—C4—C5	120.7 (4)	C28—C27—C26	120.8 (4)
C3—C4—H4	119.6	C28—C27—H27	119.6
C5—C4—H4	119.6	C26—C27—H27	119.6
C6—C5—C4	118.1 (3)	C27—C28—C29	121.6 (4)
C6—C5—N3	121.7 (4)	C27—C28—H28	119.2
C4—C5—N3	120.2 (4)	C29—C28—H28	119.2
C5—C6—C7	120.8 (4)	C30—C29—C33	117.0 (4)
C5—C6—H6	119.6	C30—C29—C28	123.8 (4)
C7—C6—H6	119.6	C33—C29—C28	119.2 (4)
C6—C7—C2	122.0 (4)	C31—C30—C29	120.5 (4)
C6—C7—H7	119.0	C31—C30—H30	119.7
C2—C7—H7	119.0	C29—C30—H30	119.7
C14—C8—C9	119.2 (4)	C30—C31—C32	118.4 (4)
C14—C8—N3	122.2 (4)	C30—C31—H31	120.8
C9—C8—N3	118.4 (4)	C32—C31—H31	120.8
C10—C9—C8	120.4 (4)	N2—C32—C31	124.3 (4)
C10—C9—H9	119.8	N2—C32—H32	117.9
C8—C9—H9	119.8	C31—C32—H32	117.9
C9—C10—C11	120.6 (3)	N2—C33—C29	122.8 (3)
C9—C10—H10	119.7	N2—C33—C34	118.2 (3)
C11—C10—H10	119.7	C29—C33—C34	119.0 (3)
C13—C11—C10	118.3 (3)	N1—C34—C26	122.9 (3)
C13—C11—C12	120.8 (3)	N1—C34—C33	117.4 (3)
C10—C11—C12	120.6 (3)	C26—C34—C33	119.7 (3)
O3—C12—O4	121.3 (4)	O1—Mn1—O4^i^	129.32 (10)
O3—C12—C11	119.0 (4)	O1—Mn1—O9	92.24 (9)
O4—C12—C11	119.6 (3)	O4^i^—Mn1—O9	85.88 (9)
C11—C13—C14	121.1 (3)	O1—Mn1—N1	96.41 (10)
C11—C13—H13	119.5	O4^i^—Mn1—N1	99.52 (10)
C14—C13—H13	119.5	O9—Mn1—N1	163.23 (10)
C8—C14—C13	120.3 (4)	O1—Mn1—N2	139.57 (10)
C8—C14—H14	119.8	O4^i^—Mn1—N2	91.11 (10)
C13—C14—H14	119.8	O9—Mn1—N2	91.28 (9)
N3—C15—C16	113.1 (4)	N1—Mn1—N2	72.85 (10)
N3—C15—H15A	109.0	O1—Mn1—O2	56.65 (9)
C16—C15—H15A	109.0	O4^i^—Mn1—O2	168.03 (9)
N3—C15—H15B	109.0	O9—Mn1—O2	83.40 (8)
C16—C15—H15B	109.0	N1—Mn1—O2	89.38 (9)
H15A—C15—H15B	107.8	N2—Mn1—O2	83.85 (9)
C17—C16—C22	118.5 (4)	O1—Mn1—O3^i^	80.00 (9)
C17—C16—C15	119.0 (4)	O4^i^—Mn1—O3^i^	55.33 (9)
C22—C16—C15	122.4 (4)	O9—Mn1—O3^i^	113.53 (10)
C16—C17—C18	120.8 (5)	N1—Mn1—O3^i^	82.21 (10)
C16—C17—H17	119.6	N2—Mn1—O3^i^	134.06 (10)
C18—C17—H17	119.6	O2—Mn1—O3^i^	134.63 (9)
C19—C18—C17	121.4 (5)	C23—N1—C34	117.3 (3)
C19—C18—H18	119.3	C23—N1—Mn1	126.7 (3)
C17—C18—H18	119.3	C34—N1—Mn1	115.8 (2)
C21—C19—C18	117.9 (4)	C32—N2—C33	117.0 (3)
C21—C19—C20	119.2 (4)	C32—N2—Mn1	127.5 (3)
C18—C19—C20	122.9 (4)	C33—N2—Mn1	115.3 (2)
O11—C20—O12	122.1 (4)	C15—N3—C5	122.5 (4)
O11—C20—C19	124.7 (4)	C15—N3—C8	113.3 (3)
O12—C20—C19	113.2 (4)	C5—N3—C8	122.1 (4)
C19—C21—C22	120.2 (4)	C1—O1—Mn1	95.7 (2)
C19—C21—H21	119.9	C1—O2—Mn1	87.3 (2)
C22—C21—H21	119.9	C12—O3—Mn1^ii^	85.9 (2)
C16—C22—C21	121.0 (4)	C12—O4—Mn1^ii^	97.4 (2)
C16—C22—H22	119.5	H8A—O8—H8B	109.5
C21—C22—H22	119.5	Mn1—O9—H9B	109.3
N1—C23—C24	123.9 (4)	Mn1—O9—H9A	109.8
N1—C23—H23	118.0	H9B—O9—H9A	109.8
C24—C23—H23	118.0	C20—O12—H12	109.4

Symmetry codes: (i) *x*+1, *y*, *z*; (ii) *x*−1, *y*, *z*.

### Hydrogen-bond geometry (Å, º)

**Table:**

*D*—H···*A*	*D*—H	H···*A*	*D*···*A*	*D*—H···*A*
O8—H8*A*···O4^iii^	0.85	1.96	2.798 (4)	170
O8—H8*B*···O11^iv^	0.85	2.43	2.872 (5)	113
O9—H9*B*···O1^v^	0.85	2.09	2.745 (3)	134
O9—H9*A*···O3^vi^	0.82	2.12	2.824 (3)	144
O12—H12···O8^vii^	0.82	1.81	2.605 (4)	165

Symmetry codes: (iii) *x*+1, −*y*+3/2, *z*+1/2; (iv) −*x*+1, −*y*+1, −*z*+1; (v) −*x*+1, *y*−1/2, −*z*+1/2; (vi) −*x*, *y*−1/2, −*z*+1/2; (vii) *x*−1, *y*−1, *z*−1.
